# Supplementary material for: The patient safety curriculum: An interventional study on the effectiveness of patient safety education for Jordanian nursing students
Source: PLoS One. 2024 May 9;19(5):e0292713. doi: 10.1371/journal.pone.0292713 (PMC11081213; doi:10.1371/journal.pone.0292713)
Supplement: Attachment — Submitted filename: Response to Reviewers.docx [file pone.0292713.s003.docx]

**Response to Editor and Reviewers**

**Dear Academic Editor and respected reviewers,**

**Thank you for the constructive comments and the effort of reviewing to enhance the manuscript. We make every effort to follow the instructions and consider each point you indicated in your review comments. See below our responses and the amendments in the track changes file uploaded.**

**We hope this will satisfy the highly respected reviewers’ recommendations and requirements of the Journal and we look forward for a decision of manuscript acceptance too.**

**Kind regards**

**Corresponding author**

**Ahmad Al-Nawafleh**

First: reply to the Editor comments

Dear Academic Editor,

Thank you for your effort and time. In the following, you will find our reply to each point you raised regarding the manuscript, and we amended it in the manuscript too. This will be identified through track changes file and kept as accepted changes in the file of the “Manuscript”.

Thank you again.

1. Please ensure that your manuscript meets PLOS ONE's style requirements, including those for file naming. The PLOS ONE style templates can be found at 
https://journals.plos.org/plosone/s/file?id=wjVg/PLOSOne_formatting_sample_main_body.pdf and 
https://journals.plos.org/plosone/s/file?id=ba62/PLOSOne_formatting_sample_title_authors_affiliations.pdf

**Manuscript edited according to PLOS ONE’s style Requirements.**

2. We note you have included a table to which you do not refer in the text of your manuscript. Please ensure that you refer to Table 1 in your text; if accepted, production will need this reference to link the reader to the Table.

**Amended: Table 1 referred to in the text.**

3. Please include captions for your Supporting Information files at the end of your manuscript, and update any in-text citations to match accordingly. Please see our Supporting Information guidelines for more information: <http://journals.plos.org/plosone/s/supporting-information>.

**Supporting Information**

**Supporting information is available from the journal server as extra file under the name: (Suplimentary documments.rar).**

4. Please review your reference list to ensure that it is complete and correct. If you have cited papers that have been retracted, please include the rationale for doing so in the manuscript text, or remove these references and replace them with relevant current references. Any changes to the reference list should be mentioned in the rebuttal letter that accompanies your revised manuscript. If you need to cite a retracted article, indicate the article’s retracted status in the References list and also include a citation and full reference for the retraction notice.

**Reference list reviewed and checked; updated according to Journal recommendations.**

Second: Reply to Reviewers’ Comments

Dear reviewers,

Thank you for your effort and time. In the following, you will find our reply to each point you raised regarding the manuscript, and we amended it in the manuscript too. This will be identified through track changes file and kept as accepted changes in the file of the “Manuscript”.

Thank you again.

Reviewer #1: It would be better to cover most of the aspect of the Multi-professional Patient Safety Curriculum Guide published by WHO in this research.

**We agree with you. However, the constraints which we indicated in the manuscript did not allow for this. We hope that we will secure enough funds in future to support a study of all aspects.**
Better to add more evidence on Literature review related to errors and patient safety.
Rearrange references: **Thank you: Done as requested.**

Reviewer #2:
Firstly, could you emerge introduction, background, and justification of the study in one part to keep the harmony of this work

**Thank you, we emerged introduction, background, and justification of the study in one part.**
Secondly, could you provide more information about the intervention

**The Intervention is added in abstracted manner. However, detailed intervention already exists within the guide published by the WHO in its guide for the evaluation of the curriculum which cited in the text too.**

Could you provide more information about the psychometric properties of the instrument that you used

**We indicated this is a tool already prepared by the WHO and tested in several places over the world including some Arab countries. This indication is added to the manuscript in page 9 under the section “study tool” in the first paragraph.**
Could you expand the discussion part to include more information about the previous study?.
No need for literature part, just used it in the discussion to compare you results with previous studies.

**We agree with you that we may delete the literature review section. However, the other reviewer indicated to expand the literature review. Therefore, we would say: more about the previous studies already exist within the literature review section that is why we kept it in the manuscript.**
Good luck
